# Cardiac rehabilitation performance predicts 1‐year major adverse cardiovascular events

**DOI:** 10.1002/clc.23890

**Published:** 2022-07-29

**Authors:** Robert Naami, Edmund Naami, Tamer Omari, Sophia Gordon Lowi, Sharon Shalom Natanzon, Vivek Patel, Addee Lerner, Ehud Rozner, Yoav Turgeman, Ofir Koren

**Affiliations:** ^1^ Internal Medicine, University Hospitals Cleveland Medical Center, Case Western Reserve University School of Medicine Cleveland Ohio USA; ^2^ School of Medicine University of Illinois Springfield Illinois USA; ^3^ Emek Medical Center Afula Israel; ^4^ Cedars‐Sinai Medical Center, Smidt Heart Institute Los Angeles California USA; ^5^ David Geffen School of Medicine University of California (UCLA) Los Angeles California USA; ^6^ Technion Israel Institute of Technology, Bruce Rappaport Faculty of Medicine Haifa Israel

**Keywords:** binary cursive partition model, cardiac rehabilitation, cox regression analysis, CR‐score, duration, MACE, outcome, performance score, speed of work, training device, workolad

## Abstract

**Background:**

Cardiac Rehabilitation is an essential following major adverse cardiovascular events however there is no current data correlating rehab performance to long term outcomes.

**Hypothesis:**

Patient exercise performance during cardiac rehabilitation reliably predicts future cardiovascular events.

**Methods:**

We conducted a single‐center study of 486 consecutive patients who participated in a CR program between January 2018 and August 2021. We assessed patient performance using a novel index, the CR‐score, which integrated duration, speed of work, and workload conducted on each training device (TD). We used a binary recursive partition model to determine the optimal thresholds for cumulative CR score. We used Cox regression analysis to assess the mortality rate among patients who developed MACE (“study group”) and those who did not ("control group”).

**Results:**

Among 486 eligible patients, 1‐year MACE occurred in 27 (5.5%) patients and was more common in patients with prior cerebrovascular accident or transient ischemic attack (14.8% vs. 3.5%, *p* < .001). Age, gender, comorbidities, heart failure, and medical treatment did not significantly affect the outcome. The median cumulative CR score of the study group was significantly lower than the control group (595 ± 185.6 vs. 3500 ± 1104.7, *p* < .0001). A cumulative CR‐score of ≥1132 correlated with the outcome (98.5% sensitivity, 99.6% specificity, 95% CI: 0.985−0.997, area 0.994, *p* < .0001). Patients older than 55 with a cumulative CR score of <1132 were at particularly high risk (OR: 7.4, 95% CI: 2.84−18.42) for 1‐year MACE (log‐rank *p* = .03).

**Conclusion:**

Our proposed CR‐score accurately identifies patients at high risk for 1‐year MACE following the rehabilitation program. Multicenter validation is required.

**Abbreviations**
ACS: acute coronary syndrome
CABG: coronary artery bypass graft surgery
CR: cardiac rehabilitation
CVA: cerebrovascular accident
MI: myocardial infarction
TD: training device
TIA: transient ischemic attack
VHD: valvular heart disease

## 1 INTRODUCTION

The American College of Cardiology and the American Heart Association currently consider cardiac rehabilitation (CR) a class I indication for a multitude of cardiac conditions.^1^ These include acute coronary syndrome, percutaneous coronary interventions (PCI), coronary bypass grafting, valvular surgery, and heart failure with reduced ejection fraction. CR requires a multidisciplinary team of providers (i.e., nurses, trainers, dieticians, physicians) and is traditionally divided into three phases.^2^, ^3^, ^4^, ^5^, ^6^ Phase I refers to inpatient rehabilitation during the hospitalization while Phases II and III refer to exercise training following discharge. Only in Phase II does the patient follow the structured exercise regimen of the rehabilitation process.^7^

CR has been shown to improve quality of life, reduce rates of readmission, and improves overall cardiovascular mortality.^8^, ^9^, ^10^, ^11^ The beneficial effects of CR are related to its physiologic effects on the body. The endurance training favorably affects hemodynamic function, vascular tone, and exercise capacity.^12^, ^13^, ^14^ In addition, CR programs provide nutritional support and smoking cessation counseling, as well as management of comorbid conditions such as hypertension, dyslipidemia, and diabetes.^15^, ^16^, ^17^

Despite growing evidence supporting the health benefits of CR, limited effort has been directed toward quantifying patient performance during rehabilitation and how this may affects outcomes. In this study, we sought to determine the relationship between exercise volume during CR and health outcomes using a novel scoring system.

### 1.1 Study design and patients' selection

We conducted a single‐center retrospective study of 516 consecutive patients who participated in a CR program at Emek Medical Center in Israel between January 2018 and August 2021. The CR program is a twice a week, 3‐month government‐funded program following recent acute coronary artery syndrome, PCI, chronic stable angina, congestive heart failure, coronary artery bypass surgery, valvular surgery, and cardiac transplantation. A multidisciplinary team consisting of cardiologists, nurse practitioners, physiotherapists, and nutritionists assessed physical performance and follow‐up progression. Each CR session performed on four different training‐devices (TD): the treadmill, elliptical, bicycle, and handcycle.

Patients were excluded from the study if they did not complete at least 80% of the program, had missing medical records or were lost to follow‐up.

### 1.2 Data collection

Demographic, procedural, and follow‐up data entered prospectively by a dedicated team and extracted using the Clalit electronic records systems (Chameleon, Ofek).

### 1.3 Ethics

The study was approved by the Emek Medical Center institutional review board, which also waived the requirement to obtain informed consent due to the study's retrospective nature.

### 1.4 Definitions

We defined outcome as the incidence rate of major cardiovascular events (MACE) during 1‐year following the CR program. MACE defined as the cumulative events of death, heart failure hospitalizations, coronary catheterizations, and hospitalizations due to cerebrovascular accident (CVA) or transient ischemic attack (TIA).

### 1.5 Statistical analysis

Continuous variables were compared between the study group and the control group using the two‐tailed student's *t*‐test or Mann−Whitney *U* test and presented as mean ± standard deviation or median and interquartile range, respectively. Categorical variables were expressed as frequencies and percentages and compared using the *χ* ^2^ or Fisher exact test.

Multivariable linear logistic regression using forward conditional method was performed using the minimum Akaike Information criteria for variable selection using all CT variables with *p* < .05 in the univariate analysis as candidates. We constructed the CR score using the integration of statistically significant variables (work time, incline, workload, and speed) found by the logistic regression and their contributions to predicting outcome. The potential of collinearity interactions among the independent variables was assessed using the variance inflation factor (VIF) and the tolerance value.

We assessed accuracy by computing receiver operating curves (ROC) and reported the ROC area (concordance statistic C) and the sensitivity and specificity at maximum accuracy, where accuracy is defined as (sensitivity + specificity)/2. In addition to the logistic models, we used a binary recursive partition (classification tree) model to determine the optimal thresholds for cumulative CR score and predictor of 1‐year MACE.

Kaplan−Meyer survival analysis was performed to assess differences in mortality rate among the two groups at 1‐year follow‐up. Hazard ratio (HR) was calculated using descriptive analysis in SPSS and linear regression models. Two‐sided *p* values were considered significant if they were less than .05. All statistical analyses were performed using JMP version 15.2.0 (SAS Institute) and SPSS statistical package, version 24.0 (SPSS Inc.).

### 1.6 CR score assessment

The CR‐score was formulated using the probability produced in the logistics regression by applying the multiplication of the exercise time, speed of work, incline percent (for the treadmill and the elliptical TDs), and the workload (for the bicycle and the handcycle TDs). The cumulative CR‐score is the summation of scores from each session of the program.

## 2 RESULTS

### 2.1 Study population

A total of 516 CR patients enrolled in our center from 2018 to August 2021. Twenty‐nine patients were excluded from the study for the following reasons: 8 patients failed to complete at least 80% of the program, 15 patients had incomplete CR records, and 5 patients lost to follow‐up.

**Figure 1:**
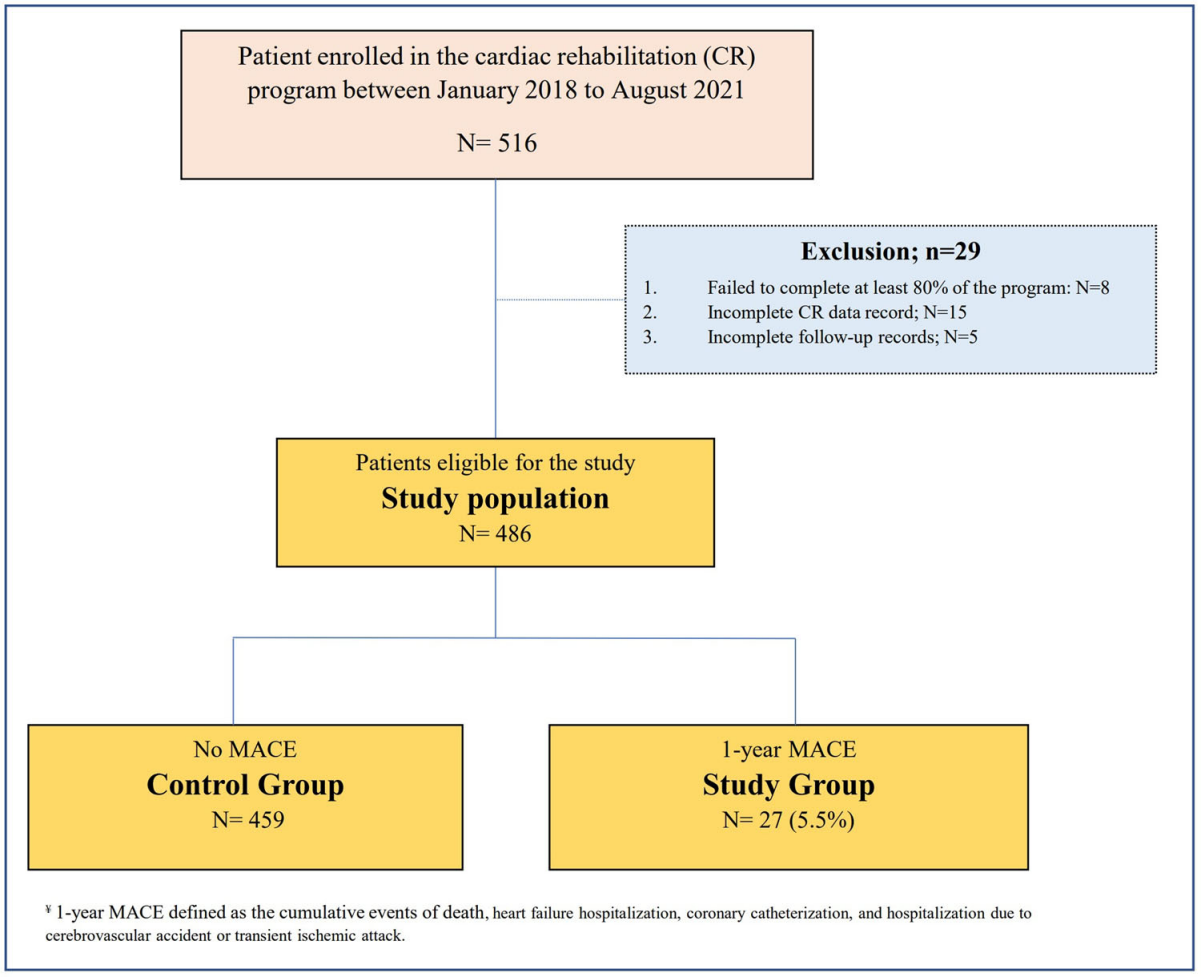
Study design. CR, cardiac rehabilitation; MACE, major adverse cardiovascular events.

Among 486 eligible patients, MACE during 1‐year of follow‐up occurred in 27 (5.5%) patients (“Study group”). We compared 459 patients (“control group”) that had no MACE to the study group (Figure 1).

### 2.2 Patients baseline characteristics

The baseline characteristics are shown in Table 1. Among the total eligible patients, the occurrence of MACE during follow‐up was more common in patients with prior CVA or TIA (14.8% vs. 3.5%, *p* < .0001) and heart failure (22.2% vs. 8.5%, *p* = .03). The CR score was correlated with the occurrence of MACE for each of the TD as well as the cumulative score (*p* < .0001 for all). A lower cumulative CR‐score was associated with worse outcomes (595.0 ± 185.63 vs. 3500.0 ± 1104.76, IQR: 217 vs. 1372, *p* < .0001) (Table 1).

**Table 1:** Patients' characteristics, procedural data, and outcome of the study groups

	Total study population	MACE	No MACE	*p* Value
	(*N* = 486) (%)	(*N* = 27) (%)	(*N* = 459) (%)	
Age (years), median ± SD (IQR)	68.4 ± 9.5 (15)	70.0 ± 8.19 (15)	64.0 ± 10.97 (16)	.416
Female sex, *n* (%)	84 (17.3)	4 (14.8)	80 (17.4)	.885
BMI, *n* (%)	23.0 ± 3.01 (8)	23.0 ± 2.96 (11)	23.0 ± 3.51 (6)	.893
Smoking, *n* (%)	139 (28.6)	7 (25.9)	132 (28.8)	.830
Hypertension, *n* (%)	331 (68.1)	20 (74.1)	311 (67.8)	.671
Lipid lowering therapy, *n* (%)	349 (71.8)	19 (70.3)	330 (71.9)	.451
Hyperlipidemia, *n* (%)	368 (75.7)	18 (66.7)	350 (76.3)	.255
Diabetes mellitus, *n* (%)	77 (15.8)	3 (11.1)	74 (16.1)	.785
Heart failure, *n* (%)	45 (9.3)	6 (22.2)	39 (8.5)	.030
Atrial fibrillation, *n* (%)	36 (7.4)	4 (14.8)	32 (7.0)	.130
Chronic kidney disease^a^, *n* (%)	4 (0.8)	2 (7.4)	2 (0.4)	.017
Coronary artery disease, *n* (%)	204 (42.0)	15 (55.6)	189 (41.2)	.162
COPD, *n* (%)	8 (1.6)	1 (3.7)	7 (1.5)	.369
CVA/TIA, *n* (%)	20 (4.1)	4 (14.8)	16 (3.5)	<.0001
Valvular heart disease^b^, *n* (%)	11 (2.3)	1 (3.7)	10 (2.2)	.470
LVEF ≤ 35%, *n* (%)	35 (7.2)	3 (11.1)	32 (7.0)	.432
Chronic use of beta blocker, *n* (%)	265 (54.5)	19 (70.4)	246 (53.6)	.112
Chronic use of ACEI, *n* (%)	222 (45.7)	9 (33.3)	213 (46.4)	.234
Chronic use of neprilysin Inhibitors, *n* (%)	8 (1.6)	0 (0)	8 (1.7)	.631
Elliptical CR score^c^, median ± SD (IQR)	364 ± 149.82 (254)	148.0 ± 53.81 (55)	381 ± 144.60 (239)	<.0001
Handcycles CR score^d^, median ± SD (IQR)	1400.0 ± 671.72 (969)	164.0 ± 51.71 (44)	1449.0 ± 623.87 (913)	<.0001
Bicycle CR score^e^, median ± SD (IQR)	1284.5 ± 717.91 (1257)	150.0 ± 60.11 (80)	1347.0 ± 685.12 (1193)	<.0001
Treadmill CR score^f^, median ± SD (IQR)	214.50 ± 117.46 (164)	145.0 ± 51.92 (86)	221.0 ± 118.7 (161)	<.0001
Cumulative CR score^g^, median ± SD (IQR)	3453.0 ± 1243.43 (1561)	595.0 ± 185.63 (217)	3500.0 ± 1104.76 (1372)	<.0001

Abbreviations: ACEI, angiotensin‐converting enzyme inhibitor; BMI, body mass index; CABG, coronary artery bypass graft; CKD, chronic kidney disease; COPD, chronic obstructive lung disease; CR, cardiac rehabilitation; CVA/TIA, cerebrovascular accident/transient ischemic attack; LVEF, left ventricular ejection fraction; MACE, major adverse cardiovascular events.

*Baseline percent of incline starts at 1 and increase respectively (1% equal 2 in the equation).

^a^ Chronic kidney disease defined as stage ≥III by the National Kidney Foundation classification.

^b^ Of at least a moderate grade.

^c^ Elliptical CR score = work time (min) × incline* (%) × speed (m/h).

^d^ Treadmill CR score = work time (min) × incline* (%) × speed (m/h).

^e^ Handcycles CR score = work time (min) × workload (W) × speed (m/h).

^f^ Bicycle CR score = work time (min) × workload (W) × speed (m/h).

^g^ Cumulative CR score = elliptical CR + treadmill CR + handcycles CR + bicycle CR.

**Figure 2:**
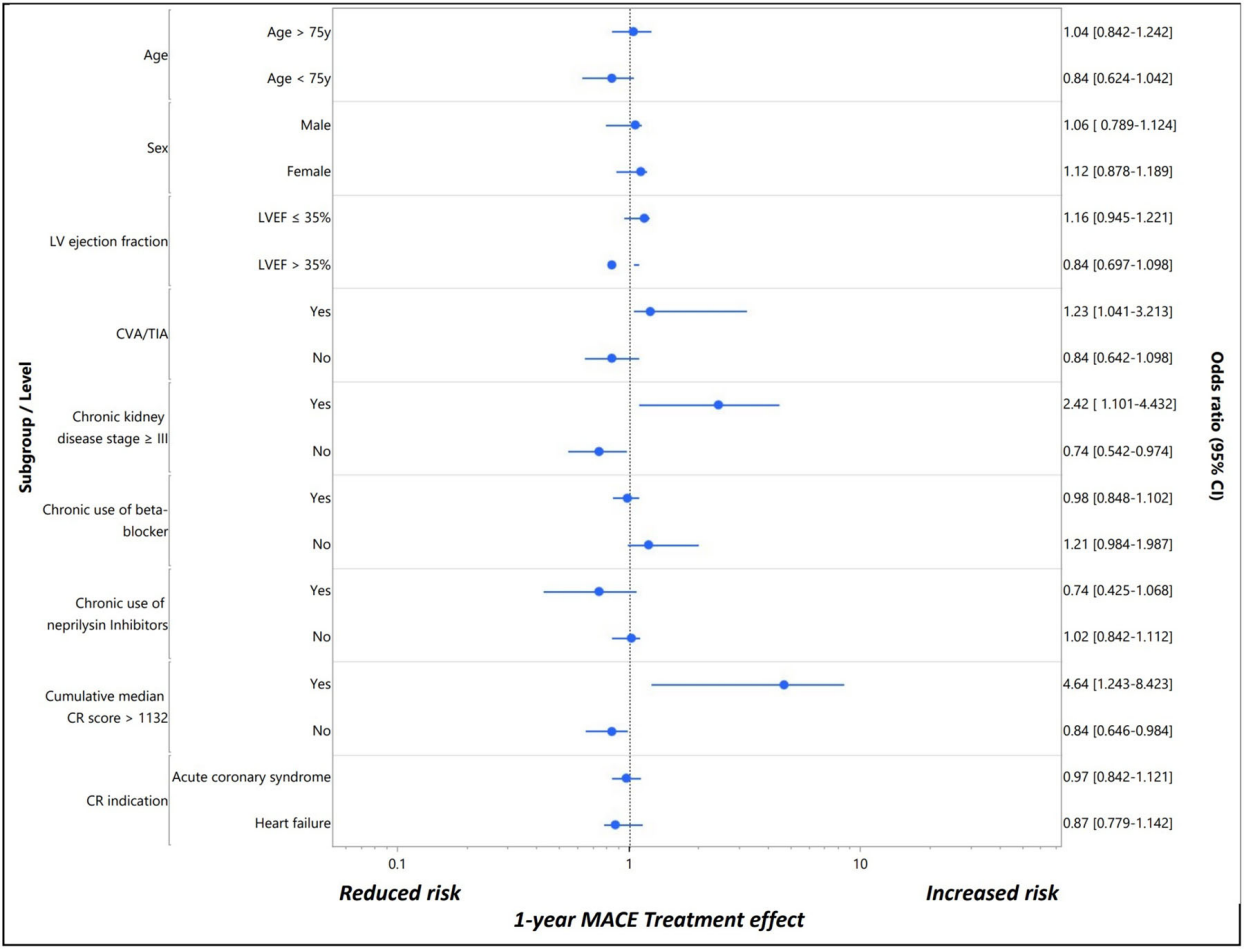
Subgroup analysis for 1‐year MACE (recursive partitioning tree). CR score, cardiac rehabilitation score; CVA/TIA, cerebrovascular accident/transient ischemic attack; LVEF, left ventricular ejection fraction; MACE, major adverse cardiovascular events.

Logistics regression analysis of age, sex, diabetes mellitus, chronic kidney disease, CVA or TIA, LV ejection fraction of ≤35%, and cumulative CR score indicated that the latter has the highest significant prediction of all (OR: 4.64, 95% CI: 1.243−8.423) with no collinearity interaction (VIF of 1.089 and tolerance of 0.918) (Table 2, Figure 2).

**Table 2:** Multivariate logistics regression analysis of MACE predictors at 1‐year follow‐up

	*p* Value	Odds ratio	95% CI
Age (years)	.319		
Female sex	.424		
BMI	320		
Smoking	.877		
Hypertension	.756		
Hyperlipidemia	.104		
Diabetes mellitus	.372		
Congestive heart failure	.111		
Atrial fibrillation	.258		
Chronic kidney disease^a^	.001	2.421	1.101−4.432
Coronary artery disease	.431		
COPD	.165		
CVA/TIA	.006	1.231	1.041−3.213
Valvular heart disease^Ω^	.285		
LVEF ≤ 35%	.097		
Chronic use of beta blocker	.126		
Chronic use of ACEI	.424		
Chronic use of neprilysin Inhibitors	.286		
Cumulative CR score^b^, ^c^	<.0001	4.64	1.243−8.423

Abbreviations: ACEI, angiotensin‐converting enzyme inhibitor; BMI, body mass index; CABG, coronary artery bypass graft; CKD, chronic kidney disease; COPD, chronic obstructive lung disease; CR score, cardiac rehabilitation score; CVA/TIA, cerebrovascular accident/transient ischemic attack; LVEF, left ventricular ejection fraction; MACE, major adverse cardiovascular events.

^a^ Chronic kidney disease defined as stage ≥III by the National Kidney Foundation classification.

^b^ Cumulative CR score represent the summation score of the following parameters: elliptical CR, treadmill CR, handcycles CR, bicycle CR.

^c^ VIF collinearity interaction of 1.089 and tolerance of 0.918.

ROC curve for 1‐year MACE using each of the TD's score used to set a threshold value encompassed the highest sensitivity and specificity for events to occur. A cumulative CR score of ≥1132 had a sensitivity of 98.5% and a specificity of 99.6% to predict outcome events (area 0.994, 95% CI: 0.985−0.997, *p* < .0001) (Supporting Information: Figure S).

Survival analysis demonstrated that patients older than 55 years of age with low cumulative CR score (less than 1132) are at particular high risk (HR: 7.4, 95% C:I 2.84−18.42) (Supporting Information: Figure S). Kaplan−Meyer survival curve indicated that MACE at 1‐year follow‐up occurred much earlier in patients with low CR score (log rank *p* = .03) (Supporting Information: Figure S).

## 3 DISCUSSION

In this study, we assessed the prognostic value of exercise performance during CR using a novel index, the CR‐score. The CR‐score value was computed using measurements of endurance through a series of exercise routines including treadmill, bicycling, elliptical, and handcycles. We demonstrated that 1‐year MACE, is associated with worse performance during CR. Moreover, CR‐score below a calculated threshold was found to be a powerful predictor of adverse outcomes, particularly among young patients.

CR is an outpatient program formulated to reduce long‐term morbidity, hospital admissions, and cardiovascular death following acute coronary syndrome, heart failure exacerbation, and cardiac surgery. Previous reports suggested favorable outcomes of those attending CR. Furthermore, recent studies provided evidence of a continuous, linear, dose−response association between CR participation and MACEs.^18^, ^19^, ^20^, ^21^, ^22^, ^23^ Possible explanations for the strong association between attendance and outcomes may include both direct effects of endurance exercise of the cardiovascular system as well as indirect benefits of physical training under medical supervision during the rehabilitation period. The former includes increased maximal oxygen uptake (VO_2_), improved endothelial function, and weight loss.^24^, ^25^ Conversely, adherence to early CR may be a surrogate of goal‐directed medical therapy compliance, general activity levels, and adherence to lifestyle choice that promote cardiac health. This includes physical exercise post‐rehab, diet, and smoking cessation.^26^, ^27^, ^28^, ^29^, ^30^, ^31^

**Figure 3:**
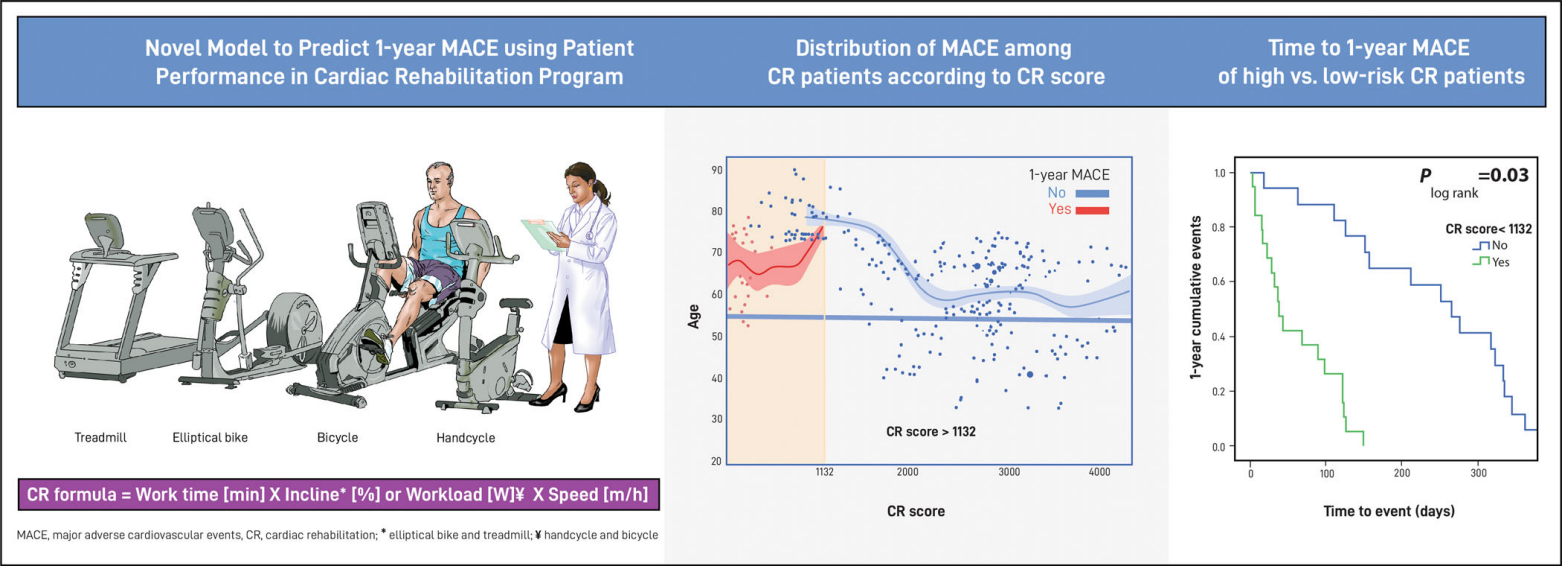
Central illustration. CR, cardiac rehabilitation; MACE, major adverse cardiovascular events.

Limited data exist regarding the prognostic utility of CR performance. Our findings are consistent with previous data suggesting the overall cardiovascular health benefits associated with rigorous endurance training following cardiovascular events (Figure 3).

Understanding the level of endurance required to promote improved outcomes can help with structuring rehabilitation programs to tailor the type of endurance training, equipment used, and duration of therapy.^32^, ^33^, ^34^, ^35^, ^36^, ^37^ Moreover, it lends quantifiable goals that patients and providers may strive to achieve together. This promotes a united and transparent front in what can be an arduous healing process.^38^, ^39^, ^40^

## 4 LIMITATIONS

The retrospective nature, the relatively small‐scaled cohort population, the low prevalence of MACE, and a single‐center‐based data analysis are the most significant factors influencing the study's strength.

Furthermore, The CR score is a novel score and by that, was not validated on a large scale with a different data base.

## 5 CONCLUSION

A patient's performance in a 3‐month supervised CR program is directly linked to the physical capacity, emotional status, and frailty that proved to influence outcomes.

Implementing the following three factors: duration, speed of work, and workload conducted on each TD in a simple formula, accurately predicts MACE at 1‐year follow‐up.

In current practice, the metabolic equivalent task (MET) scaling tool is widely used during cardiopulmonary exercise testing. It utilizes a complex ventilatory gas analysis before, during, and after the CR program and shows a strong correlation with the patient's functional capacity and future adverse cardiovascular events. MET may often be misleading, as it is influenced by several factors, including age and gender. The fixed assumption that 1 MET = 3.5 ml O_2_/kg/min has been challenged in numerous studies that indicate a significant overestimation of actual resting energy expenditure in some populations, including coronary patients, the morbidly obese, and individuals taking beta‐blockers.^41^ Our cohort included a diverse population of patients with heart failure, coronary disease, and valvular pathologies. We designed a simple‐to‐use model that focuses on participants' progression during the CR program  and does not rely upon periodic tests and patients' baseline capacity. Furthermore, the proposed model does not require specific equipment and can be adapted to any age and fragility status.

## CONFLICT OF INTEREST

The authors declare no conflict of interest.

## Supporting information

Supporting information.

## Data Availability

The data that support the findings of this study are available from the corresponding author, O. K., upon reasonable request.
