# Supplementary material for: Cardiac rehabilitation performance predicts 1‐year major adverse cardiovascular events
Source: Clin Cardiol. 2022 Jul 29;45(10):1036–43. doi: 10.1002/clc.23890 (PMC9574735; doi:10.1002/clc.23890)
Supplement: Supplementary file 1 — Supporting information. [file CLC-45-1036-s001.docx]

**Cardiac Rehabilitation Performance Predicts 1-Year Major Adverse Cardiovascular Events**

**Running title: Cardiac rehabilitation performance predicts 1-year outcome**

Robert Naami, MD^1^, Edmund Naami, MD^2^, Tamer Omari, MD^3^, Sophia Gordon Lowi, BA^3^, Sharon Shalom Natanzon, MD^4^, Vivek Patel, MS^4^, Addee Lerner, BS^5^, Ehud Rozner, MD^3^, Yoav Turgeman, MD^3,6^, Ofir Koren, MD*^3,4,6^.

^1^ Internal Medicine, University Hospitals Cleveland Medical Center, Case Western Reserve

University School of Medicine, Cleveland

^2^ School of Medicine, University of Illinois, Illinois

^3^ Emek medical center, Afula, Israel

^4^ Cedars-Sinai Medical Center, Smidt Heart Institute, Los Angeles, California

^5^ David Geffen School of Medicine, University of California (UCLA), Los Angeles, California

^6^ Bruce Rappaport Faculty of Medicine, Technion Israel Institute of Technology, Haifa, Israel

**Funding disclosures: None.**

**Author disclosures: None.**

**Conflict of Interest: None**.

Address for correspondence: Ofir Koren, MD

Cedars-Sinai Smidt Heart Institute

127 S. San Vicente Boulevard, Advanced Health Sciences Pavilion

Third Floor, Suite A3100, Los Angeles, CA 90048

E-mail: Drkorenofir@gmail.com

Tel: 972-546370014

**Word count**: 1559 (from Introduction to reference)

Number of Tables: 2

Number of Figure: 3

All authors have read and approved the manuscript

**Keywords:** Cardiac rehabilitation; Performance Score; Outcome; MACE.

**Figure legends**

**Figure 1s.** Receiver operating characteristic (ROC) of type of exercise and 1-y MACE

**Figure 2s.** Distribution of MACE among the study population based on a cumulative CR score and Age

**Figure 3s.** 1-Year mortality rate among CR score above and below 1132.


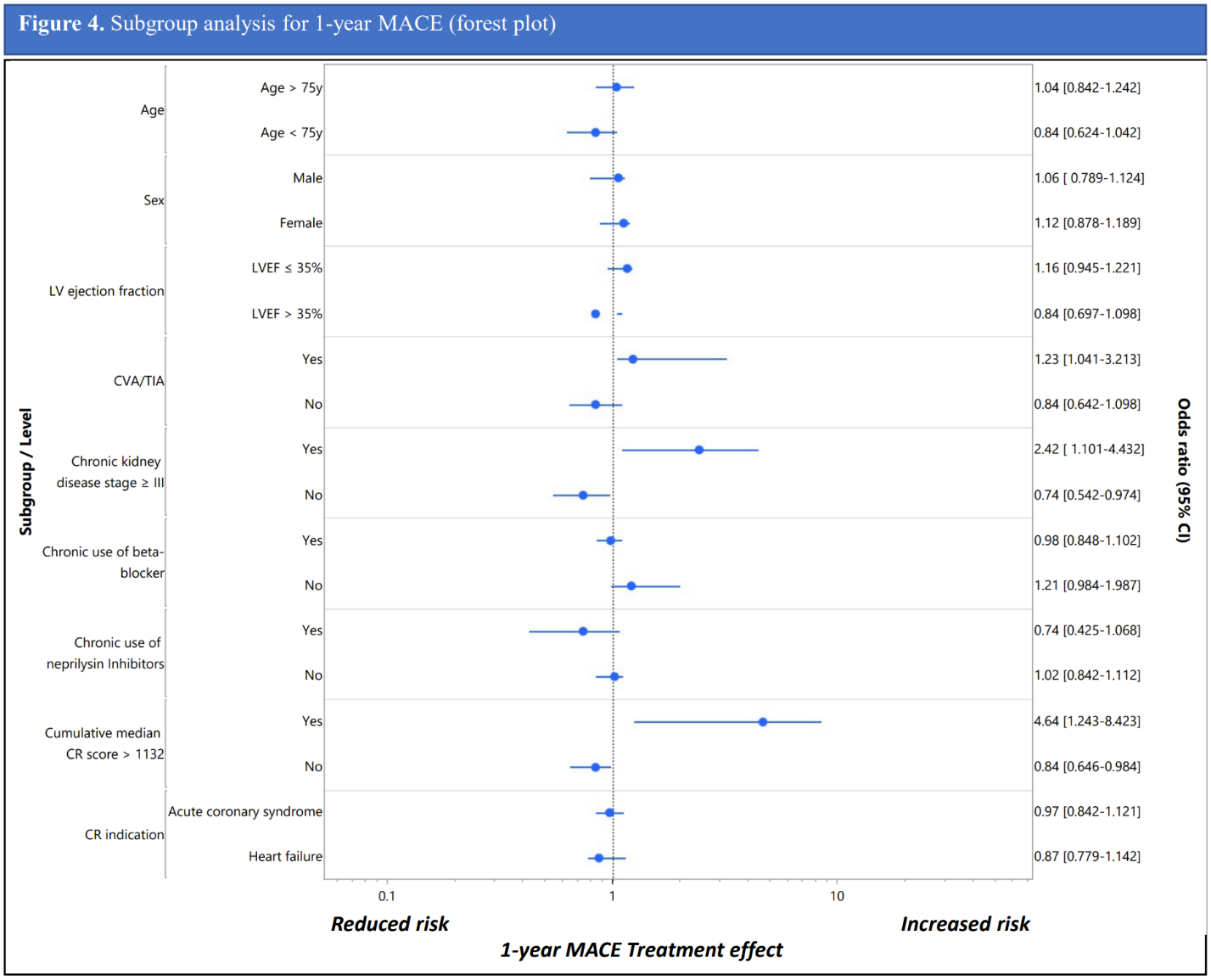
**Figure 2.** Subgroup analysis for 1-year MACE (recursive partitioning tree)

**Figure 1s.** Receiver operating characteristic (ROC) of type of exercise and 1-y MACE


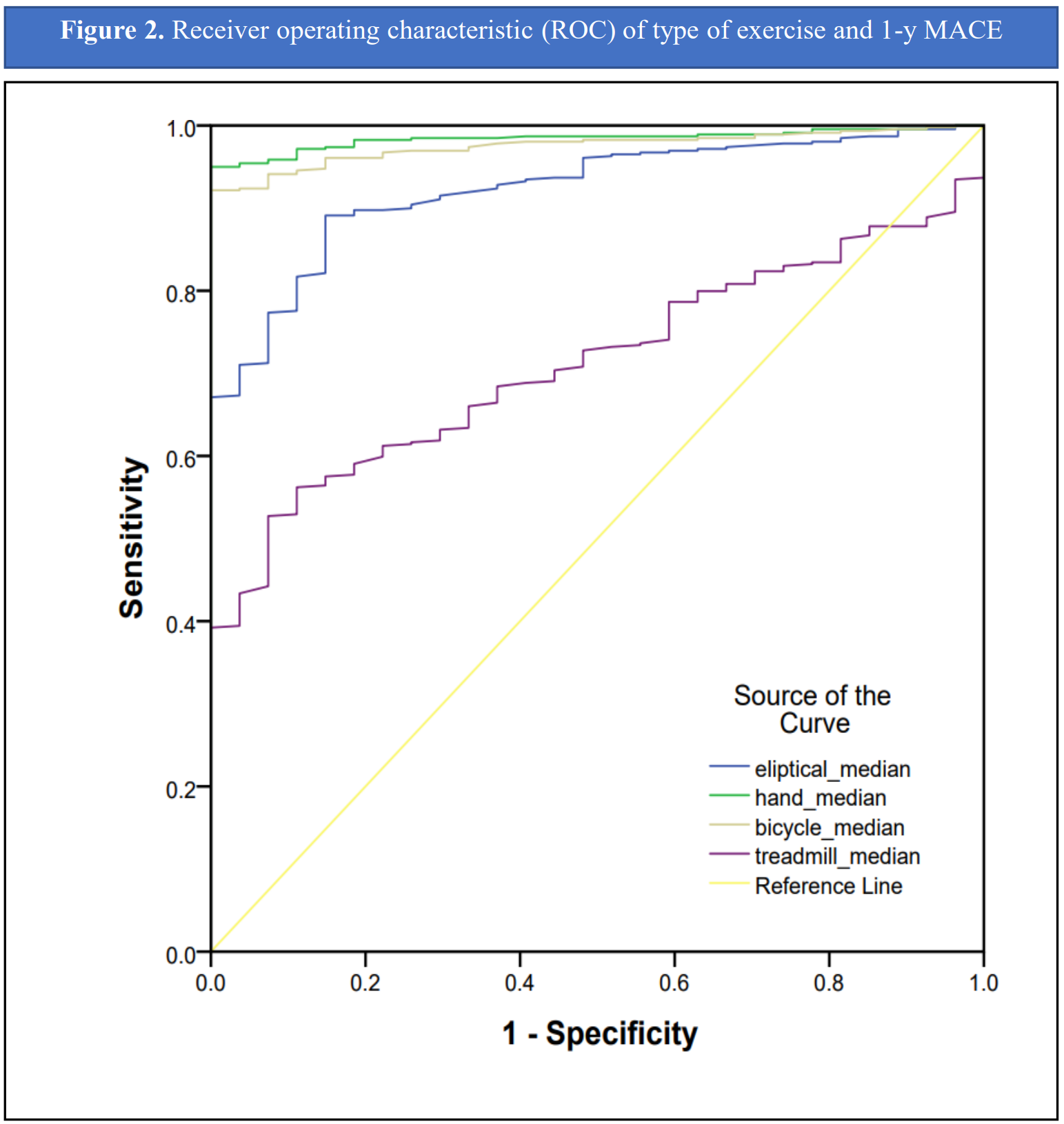


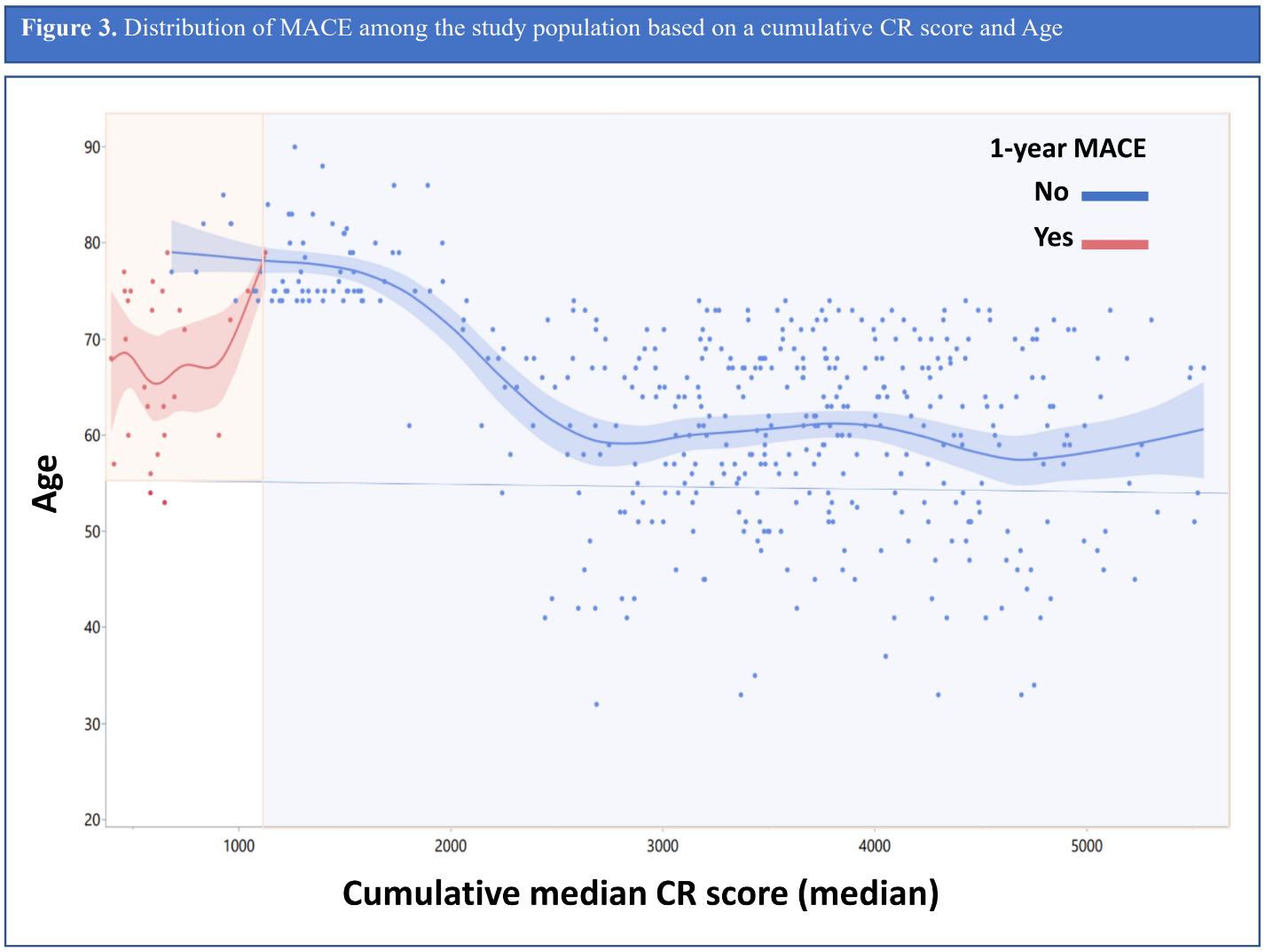
**Figure 2s.** Distribution of MACE among the study population based on a cumulative CR score and Age


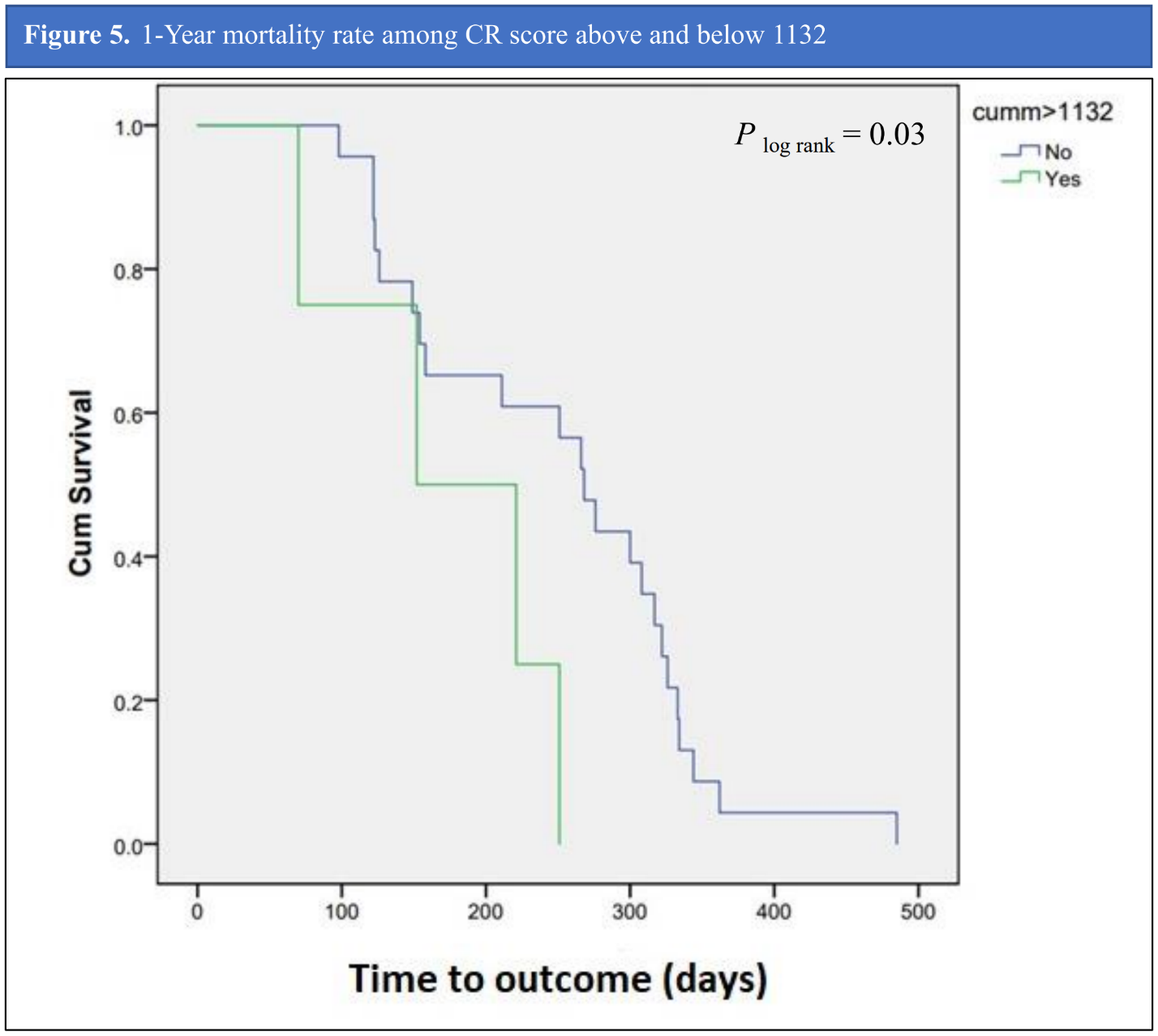
**Figure 3s.** 1-Year mortality rate among CR score above and below 1132.
